# Individual polyphenol metabotypes and longevity: microbial biotransformation and personalized nutrition interventions

**DOI:** 10.3389/fnut.2026.1913308

**Published:** 2026-08-19

**Authors:** Gözde Çalışkan Akımal, Özlem Baran, Meryem Saban Güler, Beraat Dener, Bence Raposa, Duygu Aǧagündüz

**Affiliations:** 1 Department of Nutrition and Dietetics, Faculty of Health Sciences, Muṣ Alparslan University, Mus, Türkiye; 2 Department of Nutrition and Dietetics, Faculty of Health Sciences, Lokman Hekim University, Ankara, Türkiye; 3 Department of Nutrition and Dietetics, Faculty of Health Sciences, Batman University, Batman, Türkiye; 4 Department of Nutrition and Dietetics, Faculty of Health Sciences, Bingöl University, Bingöl, Türkiye; 5 Midwifery and Health Visiting, Faculty of Health Sciences, Institute of Basics of Health Sciences, University of Pécs, Pécs, Hungary; 6 Department of Nutrition and Dietetics, Faculty of Health Sciences, Gazi University, Ankara, Türkiye

**Keywords:** biotransformation, longevity, microbiota, polyphenol, polyphenol metabotypes, urolithin

## 1 Introduction

Polyphenols are plant-derived secondary metabolites that determine the organoleptic and nutritional characteristics of plant-based foods (1). Owing to their antioxidant, anti-inflammatory, antithrombotic, antidiabetic, and anticancer properties, they reduce the risk of age-related diseases and support healthy aging (2, 3). Aging is a complex process characterized by multiple biological mechanisms, including genomic instability, epigenetic alterations, telomere shortening, impaired proteostasis, and cellular senescence (4). Polyphenols may modulate these processes by suppressing inflammatory signaling, supporting telomere stability, and regulating energy homeostasis (3, 5, 6).

The biological effects of polyphenols largely depend on their biotransformation by the gut microbiota. Most dietary polyphenols reach the colon, where they are converted into bioactive metabolites with greater bioavailability (7, 8). Interindividual differences in gut microbiota composition contribute to the emergence of distinct polyphenol metabotypes and variable responses to dietary polyphenols (3, 9). Age-related alterations in the gut microbiota may further influence these metabolic processes and contribute to individual differences in healthy aging outcomes (10, 11). The aim of this review is to examine the relationship between individual polyphenol metabotypes and longevity, with a particular focus on microbial biotransformation processes and their implications for personalized nutrition.

## 2 Gut microbiota and polyphenol biotransformation

Polyphenols are structurally complex bioactive compounds; however, their bioavailability is low, with only 5–10% being absorbed in the small intestine due to their glycosylated forms and high molecular weight. The majority (90–95%) passes into the colon, where it undergoes metabolism by the gut microbiota (7, 12).

A bidirectional interaction exists between polyphenols and the gut microbiota. Polyphenols modulate microbial composition by suppressing pathogens and supporting beneficial populations through prebiotic-like effects (13). Conversely, the gut microbiota catabolizes these compounds into more readily absorbed and bioavailable active metabolites (8).

### 2.1 Effects of polyphenols on the gut microbiota

The expanded prebiotic concept includes compounds selectively metabolized by host-associated microorganisms that promote beneficial physiological effects, such as omega-3 fatty acids, resistant dextrins, amino acids, and (poly)phenols (13, 14). Through selective bacterial fermentation, (poly)phenols like quercetin, resveratrol, and catechins exert prebiotic-like effects (15). For instance, consuming flavonoid-rich apples reduces inflammatory biomarkers and favorably alters microbiota composition (16). Consumption of pomegranate juice containing high levels of ellagic acid and ellagitannins has been shown to promote the growth of Lactobacillus and Enterococcus species (17), while cocoa flavan-3-ol-rich dark chocolate elevates beneficial bacteria, including *Faecalibacterium prausnitzii* and *Flavonifractor plautii* (18).

### 2.2 Effects of the gut microbiota on polyphenols (biotransformation)

Polyphenols serve as important substrates metabolized by the gut microbiota (3). Colonic bacteria undergo enzymatic biotransformation to convert glycosylated or high-molecular-weight polyphenols into lower molecular weight phenolic acids with higher bioavailability (19).

**Figure 1:**
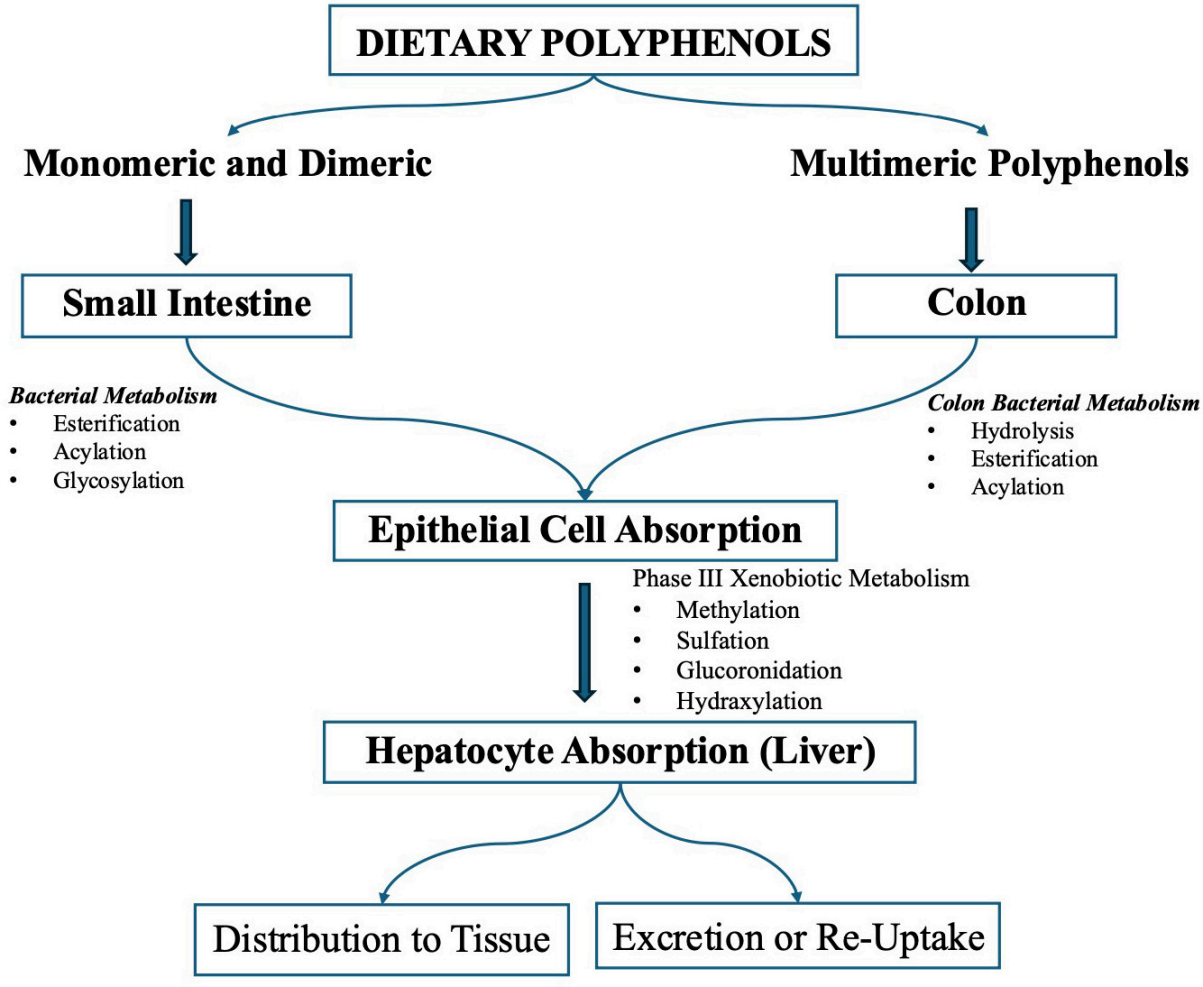
Absorption, metabolism, and gut microbiota-mediated biotransformation of dietary polyphenols. Adapted from Pasinetti et al. (19).

Monomeric and dimeric polyphenols undergo covalent modifications like acylation and esterification by bacterial enzymes in the upper gastrointestinal tract. Subsequently, they undergo phase II metabolism within enterocytes via methylation, sulfation, hydroxylation, and glucuronidation, forming O-glucuronide or O-sulfate derivatives (19, 20). In contrast, high-molecular-weight multimeric polyphenols reach the colon directly, where bacteria hydrolyze them into absorbable metabolites such as urolithins, caffeic acid, and ferulic acid derivatives (19, 21). These metabolites undergo further phase II metabolism in the liver before entering systemic or enterohepatic circulation (19, 20) (Figure 1).

Polyphenol biotransformation also contributes to intestinal homeostasis by influencing microbial metabolites involved in gut metabolism, including short-chain fatty acids (SCFAs), trimethylamine N-oxide, dopamine, bile acids, and lipopolysaccharides (7). Microbial-generated polyphenol metabolites are considered “postbiotics” owing to their favorable stability and safety profiles (22). For example, urolithin A exhibits anti-inflammatory and mitochondrial-supportive effects, whereas dihydroresveratrol demonstrates higher bioavailability (22). Furthermore, certain flavonoid metabolites benefit cardiovascular and intestinal health (23).

Nevertheless, interindividual variations in gut microbiota composition result in marked differences in the metabolism of polyphenols, resulting in distinct “(poly)phenol metabotypes” within the population (3, 9).

## 3 Individual polyphenol metabotypes

### 3.1 Gut microbiota and polyphenol metabolism

Inter-individual variability in polyphenol metabolism is largely influenced by differences in gut microbial composition and metabolic capacity (9, 24). Consequently, individuals exposed to similar amounts of dietary polyphenols may exhibit markedly different physiological responses (24, 25). Although factors such as age, sex, genotype, and physiological status contribute to this variability, gut microbiota composition and functionality are considered major determinants of polyphenol bioavailability and biological responses (9, 24, 26, 27).

The concept of “polyphenol metabotypes” emerged from the observation that individuals differ considerably in their microbial capacity to transform dietary polyphenols into bioactive metabolites. These metabotypes are generally defined by the microbial metabolites produced by individuals and their associated microbial functions (26). Importantly, the classification of metabotypes remains debated. Some researchers define metabotypes qualitatively, distinguishing individuals as producers or non-producers of specific metabolites, whereas others propose quantitative approaches based on metabolic production gradients or excretion levels (24, 27). Collectively, current producer/non-producer classifications may oversimplify the complexity of microbial polyphenol metabolism. Representative examples of currently identified polyphenol metabotypes are summarized in Table 1.

**Table 1:** Major polyphenol metabotypes, key metabolites, and reported microbial taxa.

Polyphenol class/compound	Identified metabotypes	Key metabolites	Reported microbial taxa	References
Isoflavones	Equol and ODMA producers/non-producers s	Equol, ODMA	*Adlercreutzia equolifaciens, Slackia equolifaciens, Slackia isoflavoniconvertens, Eggerthella lenta, Bifidobacterium bifidum, B. animalis, B. longum, B. pseudolongum*	(24, 29)
Ellagitannins/Ellagic acid	UMA, UMB, UM0	Urolithins	*Gordonibacter urolithinfaciens, Gordonibacter pamelaeae, Ellagibacter isourolithinifaciens*	(24, 27, 29)
Resveratrol	LP/LNP	Lunularin	Slackia equolifaciens, Adlercreutzia equolifaciens (associated with dihydroresveratrol production)	(27, 31)
Flavan-3-ols	High/low excretors, qualitative clusters	PVLs, PVAs	*Clostridium butyricum, Flavonifractor plautii, Eubacterium ramulus, Lactobacillus plantarum, Eggerthella lenta, Adlercreutzia equolifaciens*	(27, 29, 30)

ODMA, O-desmethylangolensin; UMA, urolithin metabotype A; UMB, urolithin metabotype B; UM0, urolithin metabotype 0; LP, lunularin producer; LNP, lunularin non-producer; PVLs, phenyl-γ-valerolactones; PVAs, phenylvaleric acids. Reported microbial taxa represent microorganisms associated with the corresponding metabolic pathways or metabotypes in the literature. Polyphenol biotransformation is frequently mediated by complex microbial consortia rather than individual bacterial species.

### 3.2 Urolithin metabotypes as a model of microbial polyphenol metabolism

Urolithin metabotypes represent one of the best-characterized examples of microbiota-dependent polyphenol metabolism (28). Based on urinary metabolite profiling, individuals can generally be classified into three urolithin metabotypes: UMA, characterized by urolithin A production; UMB, characterized by the production of urolithin A together with isourolithin A and urolithin B; and UM0, in which no urolithins are produced (29).

Recent evidence suggests that urolithin metabotypes may differ in their associations with cardiometabolic health during aging. UMA has been linked to a more favorable cardiometabolic profile, whereas UMB appears to exhibit greater microbial adaptability. Studies further suggest that the distribution of the urolithin metabotype may shift with aging, with decreasing UMA prevalence and progressive increases in UMB prevalence reported in older populations (24, 27, 29). These observations indicate that aging may influence both metabotype distribution and responsiveness to dietary polyphenols through changes in gut microbial composition.

### 3.3 Beyond producer/non-producer phenotypes: emerging metabotype clusters

Metabotypes produced from daidzein, a soy-derived isoflavone, represent another example of microbiota-dependent polyphenol metabolism. Daidzein can be metabolized into equol and O-desmethylangolensin (ODMA) through distinct microbial pathways (9, 24). Accordingly, individuals are generally classified as equol producers (EP) or non-producers (ENP) based on their ability to metabolize daidzein into equol (28). However, observational and clinical studies investigating the health implications of equol metabotypes have yielded inconsistent findings (24, 29). One possible explanation is the lack of standardized criteria for defining producer vs. non-producer phenotypes, as classification often depends on analytical cut-offs and methodological differences (24).

Evidence from metabolomic studies suggests that microbial polyphenol metabolism extends beyond conventional producer/non-producer classifications. Multiple metabolic clusters have been identified, reflecting differences in the metabolism of daidzein and other polyphenols, including resveratrol, flavan-3-ols, flavanones, and avenanthramides (24, 27, 30).

Since humans rarely consume isolated polyphenols, individuals may harbor combinations or “clusters” of multiple metabotypes rather than a single metabolic phenotype (26, 31). Collectively, these findings suggest that future precision nutrition approaches may require broader characterization of microbial metabolic signatures beyond simple producer/non-producer classifications (26, 31).

## 4 Aging, inflammaging, and polyphenol responsiveness

Aging is associated with changes in gut microbiota composition, characterized by reduced microbial diversity and altered bacterial populations (10, 11, 32). These age-related microbial alterations have been linked to immunosenescence and inflammaging, both of which are associated with chronic inflammation and functional decline during aging (10, 11, 33). Because SCFAs play important roles in maintaining intestinal and immune homeostasis, reductions in SCFA-producing bacteria may contribute to inflammaging and immune dysfunction during aging (10). Age-related gut dysbiosis may contribute to neuroinflammation and cognitive decline through microbiota–brain interactions (11, 34).Importantly, microbial aging does not appear to occur uniformly across all individuals. Healthy aging has been associated with preserved microbial diversity and enrichment of beneficial taxa, including *Akkermansia, Christensenellaceae*, and butyrate-producing bacteria, in centenarians and long-lived populations (10, 11, 33). These findings indicate that differences in gut microbiota composition may partly underlie individual differences in healthy aging and longevity.

Since most dietary polyphenols undergo microbial metabolism in the colon, age-associated changes in microbiota composition and metabolic functionality are likely to influence the generation of polyphenol-derived metabolites and, consequently, the overall responsiveness to dietary polyphenols (9, 10, 24). Aging is frequently accompanied by inflammaging, a chronic low-grade inflammatory condition that persists in the absence of overt infection or acute disease (35, 36). Within this inflammatory environment, responses to dietary polyphenols may also be modified. Polyphenols possess antioxidant and anti-inflammatory properties, and several of their biological effects depend on microbial conversion into bioactive metabolites (11, 35, 36). Polyphenol interventions may promote beneficial microbial taxa while reducing inflammatory signaling and improving microbial diversity during aging (10).

Recent findings further suggest that microbiota-dependent production of bioactive metabolites may contribute to healthy aging (34, 37). Among microbiota-derived polyphenol metabolites, urolithin A has attracted particular attention because of its potential effects on mitophagy and mitochondrial function. Experimental studies suggests that urolithin A may improve mitochondrial homeostasis and neuromuscular function while reducing oxidative stress and inflammation during aging (11, 38).Because urolithin A production depends on gut microbial composition, individuals may differ considerably in their capacity to generate this metabolite, highlighting the relevance of polyphenol metabotypes in aging research (24, 27, 29). Despite these promising findings, substantial inter-individual variability remains a major challenge in translating polyphenol research into personalized nutritional strategies for healthy aging. Differences in gut microbial composition strongly influence the capacity to generate specific bioactive metabolites, contributing to heterogeneous responses following polyphenol interventions (37). Taken together, these findings highlight the potential importance of microbiota-informed personalized nutrition strategies for promoting healthy aging.

## 5 Personalized nutrition interventions for healthy longevity

Differences in metabolomic signatures and gut microbial communities help explain why individuals respond differently to foods and dietary patterns, thereby underpinning the concept of personalized nutrition (39, 40). As part of dietary strategies designed to support healthy aging, modulation of the gut microbiota has emerged as a primary target for improving metabolic health (41).

Within personalized nutrition frameworks, dietary polyphenols have been recognized as key modulators of gut microbial composition and metabolic activity, with the potential to help re-establish microbial balance under dysbiotic conditions (42). Due to the heterogeneous interindividual responses to polyphenol metabolism, individualized dietary approaches based on metabolic patterns may enhance the health-promoting effects of dietary polyphenols more effectively (43). Furthermore, it has been emphasized that clarifying the relationship between microbiome characteristics and polyphenol metabotypes is essential for the development of microbiota-based precision nutrition approaches (44).

Alongside polyphenols, dietary fiber also plays a critical role in shaping gut microbiota composition and function. Through microbial fermentation, dietary fibers support a more diverse gut ecosystem, favor the growth of beneficial bacterial communities, and stimulate the generation of SCFAs, which help maintain intestinal barrier function and attenuate chronic low-grade inflammation (45–47).

Polyphenols with prebiotic properties and fiber-rich foods provide substrates for beneficial bacteria, thereby enhancing SCFA production. In particular, soluble fibers such as inulin and beta-glucans are more readily fermented than many insoluble fiber types and are associated with more pronounced metabolic benefits (48). Moreover, supplementation with prebiotics, probiotics, and synbiotics has demonstrated significant potential in maintaining microbial balance and improving metabolic health (49).

Although accurately identifying individual nutritional requirements within the framework of personalized nutrition remains challenging, implementing dietary approaches with well-established benefits in the literature appears effective for promoting healthy aging (50). The Mediterranean dietary pattern is recognized for both its sustainability and its strong evidence base supporting positive health outcomes (51). Rich in antioxidants, anti-inflammatory compounds, minerals, and vitamins, the Mediterranean diet exerts protective effects largely attributable to its high polyphenol content (52). In addition to modulating inflammatory pathways, reducing oxidative stress, improving lipid profiles, supporting gut microbiome diversity, and enhancing insulin sensitivity, this dietary pattern has also demonstrated protective potential against neurodegeneration, cognitive decline, and other aging-related pathologies (53).

While long-term dietary patterns rich in animal fats and proteins have been associated with inflammation (54), high-quality dietary patterns characterized by abundant fiber, polyunsaturated fats, and plant-based foods are strongly linked to improvements in the systemic outcomes of aging (55). In this context, sustained adherence to evidence-based, health-promoting dietary models, such as the Mediterranean diet, has been reported to support overall wellbeing, enhance the functional capacity of the gut microbiota, and influence biological processes associated with increased longevity (43).

## 6 Conclusion and future perspectives

Polyphenols are bioactive compounds with potent antioxidant, anti-inflammatory, and antithrombotic properties and may play a role in the prevention and management of various chronic diseases. The majority of dietary polyphenols reach the colon, where they undergo metabolism by the gut microbiota.Through enzymatic biotransformation, colonic bacteria convert polyphenols into lower-molecular-weight and more bioavailable metabolites, including urolithins, caffeic acid derivatives, and ferulic acid derivatives. Recent evidence has highlighted that polyphenol-derived metabotypes, particularly urolithin metabotypes, may be differentially associated with cardiometabolic health during aging. In addition, polyphenol biotransformation contributes to the maintenance of intestinal homeostasis by influencing microbiota-related metabolic outputs, including SCFAs, bile acids, dopamine, and other microbial metabolites.

However, interindividual differences in gut microbiota composition and metabolic functionality result in substantial variability in polyphenol metabolism, leading to the emergence of distinct polyphenol metabotypes. Factors such as age, sex, genotype, and physiological status further contribute to this variability. Owing to the heterogeneous nature of metabotypes and their variability across individuals, a standardized classification system has not yet been established. While some researchers classify metabotypes as producer and non-producer phenotypes, others rely on quantitative measures of metabolite production. Beyond the conventional producer/non-producer framework, numerous metabolic clusters reflecting differences in the metabolism of daidzein, resveratrol, flavan-3-ols, flavanones, and avenanthramides have also been identified. These findings suggest that classifying individuals solely as producers or non-producers may be insufficient for future personalized nutrition strategies and that microbial metabolic characteristics should be characterized in greater detail.

Accumulating evidence suggests that microbiome-mediated polyphenol metabolism may play an important role in promoting healthy aging and longevity. Aging is a complex biological process driven by the interaction of multiple mechanisms, including genomic instability, epigenetic alterations, telomere shortening, loss of proteostasis, and cellular senescence. Polyphenols have been reported to influence the molecular pathways underlying aging by supporting proteostasis and suppressing age-related inflammatory signaling pathways. Aging is also characterized by inflammaging, a state of chronic low-grade inflammation that persists over time. In this context, polyphenol-derived metabolites, particularly urolithin A, have attracted considerable attention because of their potential to reduce oxidative stress and inflammation during aging. Healthy aging has been associated with preserved microbial diversity and the enrichment of beneficial microbial taxa. Polyphenols exert prebiotic-like effects by suppressing pathogenic microorganisms while promoting beneficial microbial populations, thereby contributing to the maintenance of a favorable gut microbiota composition.

Given the heterogeneous responses to polyphenol metabolism, microbiota-based personalized nutrition strategies have gained increasing attention. Current findings indicate that microbiota-informed personalized nutrition approaches may contribute to the improvement of metabolic health, modulation of inflammaging, and promotion of healthy aging. Although several dietary approaches thought to support gut microbiota composition and healthy aging have been proposed in the literature, the existing evidence remains insufficient to establish fully individualized nutritional strategies. Further research is needed in this complex and expanding field due to methodological heterogeneity across studies, the lack of standardized metabotype classifications, and the limited availability of long-term human studies. Future studies integrating personalized microbiome analyses, metabotype profiles, and dietary patterns may help develop individualized nutritional approaches that support healthy longevity.
